# Standardized classification schemes in reporting oncologic PET/CT

**DOI:** 10.3389/fmed.2022.1051309

**Published:** 2023-01-26

**Authors:** Vanessa Murad, Roshini Kulanthaivelu, Claudia Ortega, Patrick Veit-Haibach, Ur Metser

**Affiliations:** ^1^Molecular Imaging Division, Joint Department of Medical Imaging, University Health Network, Mount Sinai Hospital and Women’s College Hospital, University of Toronto, Toronto, ON, Canada; ^2^University Medical Imaging Toronto, University of Toronto, Toronto, ON, Canada

**Keywords:** PET, Deauville, Krenning, PROMISE, immunotherapy, head and neck cancer

## Abstract

The imaging report is essential for the communication between physicians in patient care. The information it contains must be clear, concise with evidence-based conclusions and sufficient to support clinical decision-making. In recent years, several classification schemes and/or reporting guidelines for PET have been introduced. In this manuscript, we will review the classifications most frequently used in oncology for interpreting and reporting ^18^F-FDG PET imaging in lymphoma, multiple myeloma, melanoma and head and neck cancers, PSMA-ligand PET imaging for prostate cancer, and ^68^Ga-DOTA-peptide PET in neuroendocrine tumors (NET).

## 1. Introduction

The imaging report plays a fundamental role in patient care, being the main means of communication between reporting radiologists or nuclear medicine physicians, and oncologists, surgeons and patients. In addition to describing the imaging findings in a clear and concise way, a standardized, evidence-based analysis is beneficial for clinical decision-making. Standardized reporting frameworks have been developed for the interpretation of imaging studies in radiology for various lesions/malignancies. Early reporting frameworks date to the 1980s with the development of the Breast Imaging -Reporting and Data System (BI-RADS), designed to standardize the interpretation and reporting of mammograms and to facilitate the communication of patient’s risk for developing breast cancer between the radiologist and the referring physician. Since then, the American College of Radiology has developed other RADS for the interpretation of CT colonography (C-RADS), diagnosing hepatocellular carcinoma on CT and MRI (LI-RADS), lung cancer screening with CT (Lung-RADS), classification of adnexal/ovarian masses on US and MRI (O-RADS), and the description, risk stratification and management of thyroid nodules using US (TI-RADS), amongst others (1). In recent years, there have been various classification schemes introduced into clinical use in oncologic PET reporting to standardize interpretation and reporting of PET imaging findings, minimize potential interpretive pitfalls and facilitate communication between physicians in the patient’s circle of care, crucial for therapy planning. For certain malignancies, PET risk stratification is directly translated into patient management decision; for example, the escalation or de-escalation of therapy based on early interim PET in Hodgkin’s lymphoma, or the omission of lymph node neck dissection in patients with metastatic head and neck squamous cell carcinoma following chemoradiotherapy (2, 3). In this manuscript, we will review the most frequently clinically used classification schemes for the standardized interpretation and reporting of PET imaging in various malignancies, highlighting the current evidence for their utility, with illustrative case examples.

## 2. Reporting schemes and classifications

### 2.1. Therapy response assessment with ^18^F-FDG PET

#### 2.1.1. Deauville score

**FIGURE 1:**
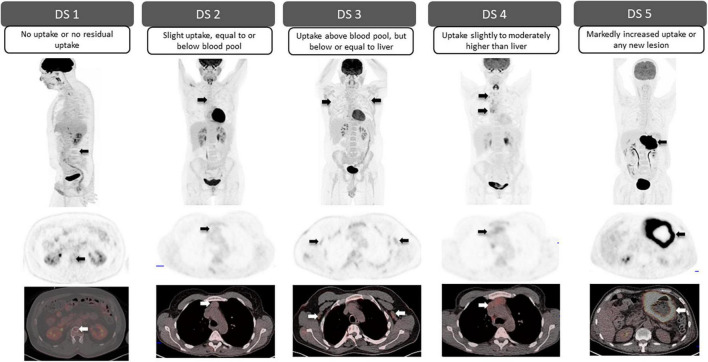
Deauville 5-point scale. Visual grading for each Deauville score (DS) according to the degree of FDG uptake in the mediastinal blood pool (MBP) and liver. Arrows point to residual lesions for each DS (**top row**: sagittal or coronal PET; **middle row**: axial PET; **bottom row**: fused axial PET/CT).

The Deauville scoring system, proposed following an international meeting in Deauville, France in 2009, was developed as a classification system that would aid the reliable description of residual fluorodeoxyglucose (FDG) avidity of lymphomas on positron emission tomography (PET) following therapy (4). Currently, it is the internationally recommended scale used in the Lugano classification for treatment response assessment in FDG-avid lymphomas, which includes most histologies with several exceptions, most notably small lymphocytic lymphoma, and some marginal zone lymphoma (5). Likewise, in recent years, the usefulness of this scoring system in the assessment of patients with multiple myeloma (MM) has also been demonstrated, specifically for determining tumor metabolic activity at both baseline staging and treatment response assessment (6). The Deauville 5-point scale uses two reference points against which FDG uptake is graded; FDG uptake in the mediastinal blood pool (MBP), determined at the level of the aortic arch avoiding the vessel walls, and FDG uptake in the liver, measured in the center of the right hepatic lobe. Visually, the FDG uptake of the lesion in query is compared with these references to assign a Deauville score (DS) as shown in (Figure 1) (7, 8).

##### 2.1.1.1. Lymphoma

^18^F-FDG-PET is a robust clinical tool for therapy response assessment in lymphoma. Its superiority over conventional imaging, high prognostic value and significant influence on treatment decisions and, therefore course of the disease, have been widely demonstrated (9–11). Whether interim or at the end of therapy, a DS of 1, 2, or 3 in the absence of FDG-avid bone marrow involvement, represents a complete metabolic response, regardless of persistent morphological lesions. In patients with a DS 4 or DS 5 partial metabolic response is suggested, when there is post-treatment decrease in FDG uptake; stable disease, when no significant interval change in metabolic activity; and progressive disease when there is increased intensity in metabolic activity and/or any new FDG-avid lymphomatous lesion (7, 8). At initial staging, it is important to recognize that the spleen, Waldeyer’s ring and thymus when involved are considered lymphatic involvement, while after therapy diffuse increased activity at these sites may be reactive and/or represent thymic rebound. Furthermore, increased splenic activity along with diffuse increased metabolic activity in the bone marrow may be seen following the administration of granulocyte-colony stimulating factor (8, 12).

**FIGURE 2:**
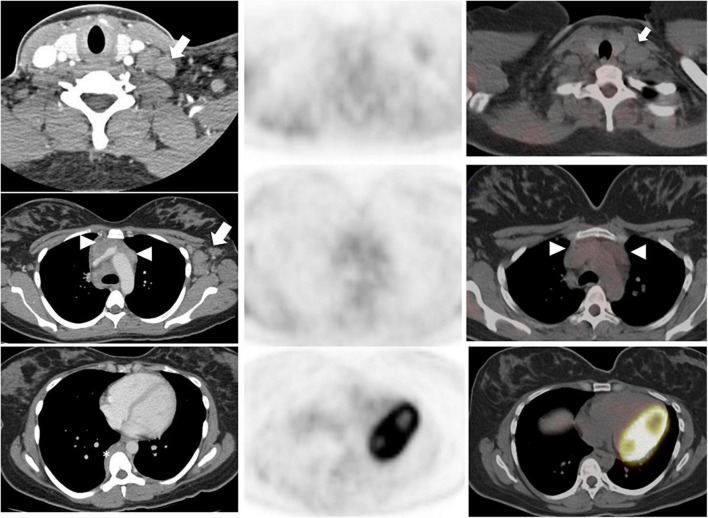
A 28-year-old woman with Stage IIB nodular sclerosing Hodgkin’s lymphoma. Baseline staging with contrast-enhanced CT **(left column)** showed multiple lymphadenopathies in the left neck, supraclavicular fossa **(arrow, top row)** and axilla **(arrow, middle row)**, an infiltrative soft tissue mass in the anterior mediastinum **(white arrowhead, middle row)**, and a paravertebral soft tissue lesion in the mid thoracic spine **(asterisk, bottom column)**. Interim ^18^F-FDG PET/CT (PET, **middle column**; fused PET/CT, **right column**) after 2 cycles of chemotherapy demonstrated complete metabolic response and significant morphologic response (**right column** fused PET/CT images); overall Deauville score, 2. A negative interim PET can support the decision to de-escalate therapy; whereas a positive PET may indicate need for therapy escalation.

A favorable interim response has been associated with progression-free survival (PFS) rates around 85–90% in patients with Hodgkin lymphoma and 70–90% in patients with non-Hodgkin lymphoma, while in patients with poor response or progression PFS rate it is around 30–40% (13). Furthermore, initial studies have suggested that PET adapted treatment strategies among patients with Hodgkin’s lymphoma, such as escalating therapy for positive interim PET in early-stage Hodgkin’s lymphoma and de – escalation of therapy (omission of Bleomycin to minimize lung toxicity) in patients with negative interim PET have resulted in favorable outcomes (Figure 2) (2, 14). At the end of treatment, the metabolic response as determined by ^18^F-FDG PET is considered a predictor of remission with a negative predictive value (NPV) as high as 90–100% (15, 16). Likewise, in the setting of relapsed or refractory lymphoma, achieving a complete metabolic response (DS 1–3) before autologous stem cell transplant (ASCT) is associated with a better long-term outcome, with 3-year PFS rates of 75% in patients with follicular lymphoma (FL) and 77% in patients with diffuse large B-cell lymphoma (DLBCL) in those who achieve complete metabolic response (DS 1–3), versus 43 and 49%, respectively, when there is only partial metabolic response or progression (DS 4 or DS 5) (17, 18).

##### 2.1.1.2. Multiple myeloma

**FIGURE 3:**
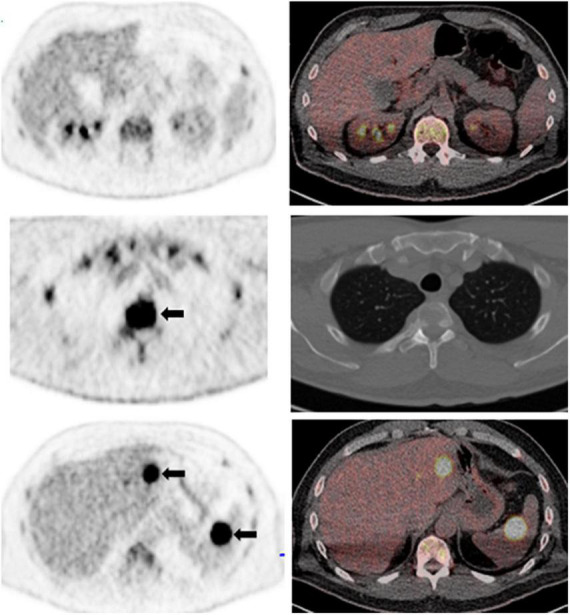
A 62-year-old man with a history of oligosecretory multiple myeloma. Whole body maximum intensity projection PET image (not shown) showed diffuse increased FDG uptake in the bone marrow above background liver (bone marrow Deaville score, 4). Representative axial PET **(left column)** and fused PET/CT images **(right column)** show diffuse increased bone marrow activity above liver **(top row)**; and intensely metabolically active skeletal lesions (T3 vertebral body; **arrows, middle row**). There are also multiple metabolically active extraskeletal focal lesions with intense FDG uptake in the liver and spleen **(arrows, bottom row)**; focal lesion Deauville score, 5.

For patients with MM, advanced imaging modalities such as MRI or ^18^F-FDG PET are recommended for evaluation and follow-up according to the International Myeloma Working Group (IMWG). PET allows the detection of both medullary and extramedullary involvement and has the ability to distinguish between metabolically active and inactive disease. However, the use of the Deauville 5-point scale was only recently introduced to determine metabolic response to therapy in MM in an objective and reproducible way, demonstrating an interobserver agreement of greater than 75% (19). At PET performed for therapy response assessment, FDG uptake in the bone marrow (BM) and in focal lesions (FLs), whether skeletal or extraskeletal, lower than that in the liver represents an independent predictive factor for prolonged progression free survival (PFS) and overall survival (OS). Therefore, a DS of 1–3 in BM and FLs has been proposed as the standardized criteria for complete metabolic response, confirming the value of the DS for assessing therapy response in patients with MM (6, 20–22). Similarly, an interval post-therapy decrease in the number and/or metabolic activity in the BM or FLs remaining above that of the liver (DS 4 or 5) is considered a partial metabolic response; no significant change in BM or FLs activity is consistent with stable disease; and metabolic progression is defined when there are new FLs consistent with myeloma compared to baseline (Figure 3).

#### 2.1.2. Hopkins criteria

**FIGURE 4:**
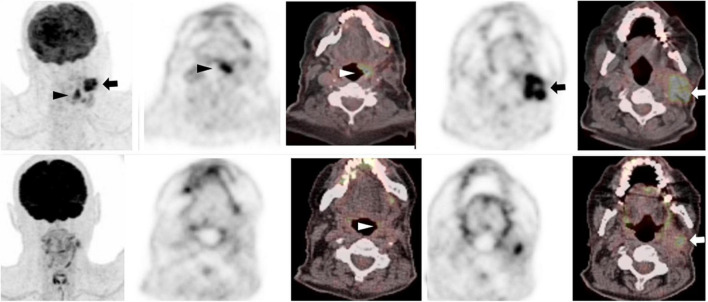
75-year-old man with left tongue base squamous cell carcinoma. Baseline ^18^F-FDG PET/CT (**top row** – zoomed MIP image left, axial PET and fused PET/CT image at level of primary tumor **(middle columns)** and neck level II lymphadenopathy **(right columns)** show an intensely metabolically active left tongue base tumor **(arrowhead)** with metabolically active metastatic left neck lymphadenopathy **(arrow)**. Follow-up PET following radical chemoradiotherapy **(corresponding images, **bottom row**)** showed complete metabolic response at the primary tumor **(white arrowhead, bottom row)**, with a persistent metabolically active left neck lymph node, Hopkins score 5, consistent with residual tumor **(white arrow, bottom row)**.

^18^F-FDG PET has good diagnostic performance in detecting tumor in patients with squamous cell carcinoma of the head and neck, with a sensitivity of 92% and specificity of 87%. However, differentiating between residual tumor and treatment related changes on PET can be challenging (23). Hopkins criteria were introduced in 2014, to standardize the interpretation of therapy response of head and neck squamous cell carcinomas to chemoradiotherapy and to better distinguish between residual tumor and therapy-related inflammatory change (24). Residual metabolic activity in the neck is described using a 5-point qualitative scale, visually correlating FDG uptake at tumor sites with metabolic activity in reference tissues; the internal jugular vein (IJV) and the liver. A score is assigned for both the primary tumor and metastatic lymph nodes, and the highest Hopkins score will guide the final interpretation (Table 1). When the uptake at the primary site or lymph nodes is less than the uptake in the IJV, a score of 1 is assigned, corresponding to a complete metabolic response. If the uptake, either in the primary site or in the lymph nodes, is greater than the uptake in the IJV but less than in the liver, it is considered a Hopkins score of 2, which represents a likely metabolic complete response. When FDG uptake at the region of interest is slightly greater than that of the liver and/or diffuse, this corresponds to a score of 3, indicating likely post radiation inflammation. However, when the uptake is moderately greater than that of the liver and focal, this represents likely residual tumor corresponding to score 4. Finally, Hopkins score 5, indicating definite residual tumor, is assigned when the uptake at the lesion is intense, markedly above liver, and focal (Figure 4). These criteria have an excellent interobserver agreement and a high negative predictive value (reported NPV of up to 95%). A recently published validation study on 259 patients with ^18^F-FDG PET following definitive radiotherapy for oropharyngeal squamous cell carcinoma showed that a positive Hopkin’s score was associated with local residual disease/recurrence rate of 45% compared to 5% when negative (Hazard ratio, 12.60; *p* < 0.001) (25) and these are also predictive of PFS and OS (26). Additionally, post-therapy the Hopkins criteria on ^18^F-FDG PET may identify clinically unsuspected residual disease in up to 19.5% of patients (24, 27).

**TABLE 1:** Hopkins 5-point score and interpretation.

Hopkins score	Definition	Type of response
1	Uptake at the primary site/lymph nodes below the uptake in the internal jugular vein (IJV)	Metabolic complete response
2	Uptake at the primary site/lymph nodes greater than the uptake in the IJV, but below than that in the liver	Likely metabolic complete response
3	Diffuse uptake at the primary site/lymph nodes, greater than the uptake at the IJV or liver	Likely postradiation inflammation
4	Focal uptake at the primary site/lymph nodes, greater than the uptake at the IJV or liver	Likely residual tumor
5	Focal and intense uptake at the primary site/lymph nodes	Definitive residual tumor

#### 2.1.3. Lymphoma response to immunomodulatory therapy criteria (LYRIC)

LYRIC was proposed in 2016 are a modification of the Lugano criteria adapted for the follow-up of patients with immune-based therapy for lymphoma, specifically checkpoint inhibitors, given the increase and availability of these new biological agents with specific properties. The “tumor flare phenomenon,” also known as pseudoprogression, associated with these therapies is characterized by rapid activation of immune cells (e.g., natural killer cells, surface molecules) and accelerated tumor necrosis, with associated increased inflammatory response (28). Based on this, there are four possible response patterns in these patients: (1) shrinkage of initial lesions, without new lesions; (2) no change or stable disease; (3) initial increase in total tumor burden with subsequent response; and (4) response despite having new lesions. The LYRIC criteria seek to modify the traditional response assessment criteria to recognize the potential flare phenomenon. In these modified criteria, a new response category termed indeterminate response (IR) was introduced, to facilitate continuation of treatment in this clinical trial setting, with a mandatory re-evaluation to confirm true therapy response. IR is defined by: (1) an increase in overall tumor burden, (2) interval development of new lesions or growth of one or more existing lesion, and/or (3) interval increase in FDG uptake at one or more lesions, without morphologic change (Table 2). For those categorized as IR on initial therapy response ^18^F-FDG PET, the same therapy is continued, and a new PET scan is obtained 12 weeks later (or earlier if clinically indicated), to reassess the response with respect to the nadir study, as shown in Table 3 (8, 29, 30). Although pseudoprogression has been documented in less than 10% of patients (31, 32) in clinical practice these criteria are very useful since they provide flexibility in therapeutic decision-making; though to date, these criteria have not been widely validated. A recently published study (33) from the Lymphoma Study Association has suggested that LYRIC is useful only for early evaluation, with a 3.4% rate of pseudo-progression observed after 4 cycles of atezolizumab, venetoclax and obinutuzumab in relapsed or refractory DLBCL and FL but none after 8 cycles of therapy.

**TABLE 2:** LYRIC criteria, definitions for indeterminate response (IR) category.

Indeterminate response (IR)	Definition
IR (1)	Increase in overall tumor burden* ≥50%, in the first 12 weeks of therapy, without clinical worsening
IR (2)	Development of new lesions or Increase ≥50% of one or more existing lesion(s) at any time during treatment, without ≥50% increase of overall tumor burden*
IR (3)	Increase in FDG uptake of one or more lesions, without any increase in size of those lesions, or new lesions

*Tumor burden: sum of the product of the perpendicular diameters of up to six measurable lesions.

**TABLE 3:** LYRIC criteria: response assessment during follow-up for IR.

Response type	Definition*
IR complete remission	Complete resolution of all lesions, with no new lesions present, no less than 4 weeks from the date first documented
IR partial remission	Decrease in tumor burden ≥50% with respect to nadir
IR stable disease	Not meeting criteria for complete or partial response, in the absence of progression
IR progressive disease	Increase in tumor burden ≥25% with respect to nadir

*All criteria should be determined in a consecutive assessment, at least 4 weeks after the date first documented.

#### 2.1.4. PET Response Evaluation Criteria for Immunotherapy (PERCIMT)

Immune-modulating therapies are becoming more available and have wider applications. Broadly speaking, there are two types of immunotherapies, passive and active. In passive immunotherapies, the goal is to enhance the patient’s immune response by targeting existing anti-tumor mechanism such as antibodies against immune checkpoints, which are the most widely used (e.g., cytotoxic T-lymphocyte associated protein 4 – CTLA-4, programmed cell death protein-1 – PD–1, programmed death-ligand 1 – PDL1). Conversely, in active therapies, the aim is to direct the immune system against tumor cells by targeting specific tumor antigens; an example of this is the use of chimeric antigen receptor (CAR) T cell therapy, where the biological agent redirects the patient’s own T cells against tumor cells (34, 35).

**FIGURE 5:**
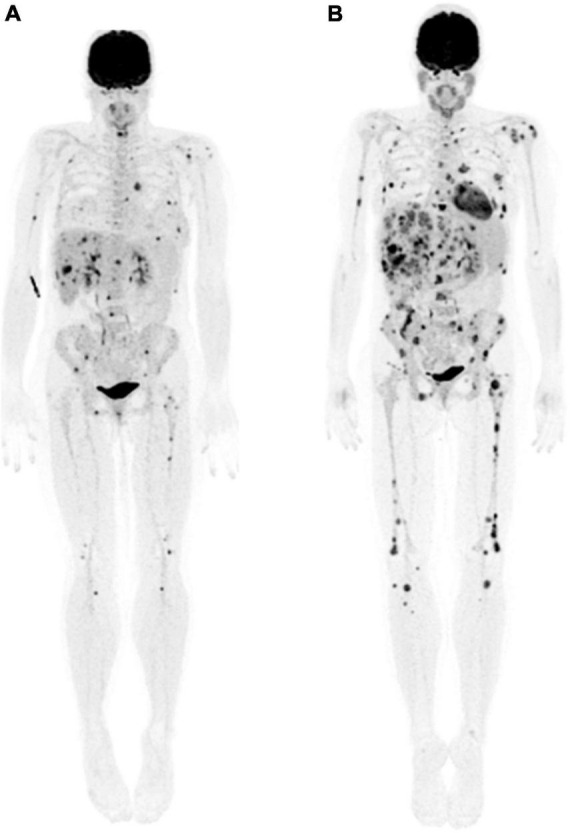
44-year-old woman with presumed stage IIIB melanoma of right buttock. **(A)** Maximum intensity projection whole body ^18^F-FDG PET image showed extensive metabolically active metastatic disease in the breasts, liver, and skeleton. **(B)** Three months following initiation of immunotherapy, follow-up maximum intensity projection whole body ^18^F-FDG PET image showed interval development of innumerable new liver, skeletal and breast lesions, most with diameter <15 mm, in keeping with progressive disease according to PERCIMT criteria.

The clinical use of immunotherapies led to the recognition of different therapy response patterns, as well as immunotherapy-related toxicities, requiring development of new response assessment measures. Various criteria were proposed in the early years of clinical implementation of immunotherapy, such as the EORTC criteria [European Organization for Research and Treatment of Cancer (36)] and the PERCIST criteria [PET Response Criteria in Solid tumors (37)] for the metabolic response assessment to immunotherapy for patients with melanoma. However, these criteria had limited predictive value of outcomes (34). Later, in 2017, PECRIT criteria (PET/CT Criteria for Early Prediction of Response to Immune Checkpoint Inhibitor Therapy) were introduced. It was recognized that during immunotherapy, the number of new lesions, rather than the FDG uptake in pre-existing tumor sites, was predictive of the clinical outcome (38, 39). Taking these observations into consideration, in 2018 the PERCIMT criteria (PET Response Evaluation Criteria for Immunotherapy) were proposed and are currently the internationally recommended criteria for the follow-up of these patients for both interim and end of therapy response assessment (40). Five categories of response to treatment are defined according to the presence of lesions, their number and size. A complete response consists in the resolution of all lesions in the absence of new FDG-avid lesions, and a partial response is considered when there is resolution of some of the FDG-avid lesions without new lesions. Disease progression is determined in the following 3 scenarios: (1) when there are 4 or more new lesions measuring less than 10 mm in their functional diameter, (2) when there are 3 or more new lesions with functional diameter greater than 10 mm, or (3) when there are 2 or more new lesions with a functional diameter greater than 15 mm (Figure 5). Stable disease is defined when there are no definitive criteria for any of the above (34). There are few studies showing the correlation of PERCIMT criteria with clinical outcomes. In the interim evaluation, PERCIMT has been shown to be more accurate in predicting the final clinical response than the EORTC criteria (40). At the end of therapy, PERCIMT has been shown be a reliable indicator of treatment failure, correlating directly with PFS and OS (39).

### 2.2. Staging with prostate-specific membrane antigen (PSMA)-targeted PET

#### 2.2.1. Prostate Cancer Molecular Imaging Standardized Evaluation (PROMISE)

The use of prostate-specific membrane antigen (PSMA)-targeted PET imaging has revolutionized the primary staging, therapy monitoring and assessment of recurrent disease in men with prostate cancer, with a significant impact on management and clinical outcomes. It is estimated that approximately 90% of prostate cancers overexpress PSMA. On PSMA-ligand PET imaging more than 90% of nodal metastases with a short axis of at least 4.0 mm can be identified (41). In primary staging, it is indicated for patients with unfavorable intermediate or high-risk disease, leading to a change in management in up to 22–28% of cases (42). In therapy response assessment, it is considered the main imaging biomarker for classifying patients as responders (stable disease, partial or complete response) or non-responders (disease progression, defined as an increase in tumor burden greater than 30%), leading to essential changes in management (43). Finally, in the biochemical recurrence setting, PSMA-PET may detect recurrence in up to 38% in patients with serum PSA under 0.5 ng/ml, and in up to 83% when serum PSA is >1 ng/ml. This results in a change in therapy in more than half of patients, including change in management intent in 30% of cases (more commonly from palliative to potentially curative intent) (44).

The Prostate Cancer Molecular Imaging Standardized Evaluation (PROMISE) classification, proposed in 2018, consists of a standardized reporting framework to organize PSMA-ligand PET/CT or PET/MRI findings into specific categories to increase diagnostic certainty in the interpretation of PSMA-targeted PET imaging and endorse the exchange of information among physicians and institutions. It is also known as molecular imaging TNM (miTNM, version 1.0) as it follows the framework of clinicopathological TNM, describing the presence, location, and extent of local prostate cancer or recurrence, as well as of pelvic and extra pelvic metastatic disease (41). Based on the degree of PSMA uptake and presence or absence of findings on conventional imaging, a categorical diagnosis is provided along with the degree of confidence: positive (consistent with, or suggestive of), equivocal or negative (unlikely, or no evidence of disease); Table 4 (45).

**TABLE 4:** Guide for interpretation of PSMA-avid lesions according to location and morphology.

Lesion location	Lesion appearance on CT/MRI	miPSMA score	Diagnosis
Prostate gland lesions	PI-RADS 5	Any	Positive
	PI-RADS 4	≧1	Positive
		0	Equivocal
	PI-RADS 3	≧2	Positive
		1	Equivocal
		0	Negative
	PI-RADS 2-1	≧2	Positive
		≦1	Negative
	MRI not available	≧2	Positive
		≦1	Negative
Prostate bed s/p radiation therapy	Intraprostatic lesion	≧2	Positive
		1	Equivocal
		0	Negative
	No intraprostatic lesion	≧2	Positive
		≦1	Negative
Prostate bed s/p prostatectomy	Soft tissue lesion	≧1	Positive
		0	Negative
	No soft tissue lesion	≧2	Positive
		≦1	Negative
Lymph nodes	>8 mm or enhancement	>1	Positive
		0	Negative
	Unremarkable pelvis/retroperitoneum	>1	Positive
		0	Negative
	Unremarkable other regions	≧2	Positive
		≦1	Negative
	Common pitfall or non-prostate cancer malignancy suspected	3	Positive
		2	Equivocal
		≦1	Negative
Bone and visceral organs	Suspicious	≧1	Positive
		0	Negative
	Equivocal	≧2	Positive
		≦1	Negative
	No lesion/single focus	3	Positive
		2	Equivocal
		≦1	Negative
	No lesion/multiple foci	≧2	Positive
		≦1	Negative
	Benign lesion or non-PCa tumor	3	Positive
		≦2	Negative

Adapted from Eiber et al. (41).

**FIGURE 6:**
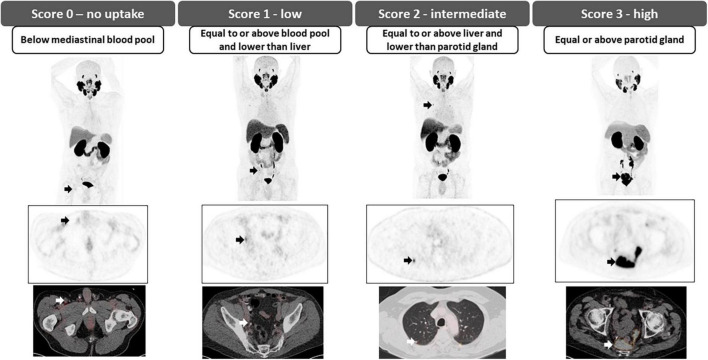
miPSMA expression score. Examples for the various miPSMA scores are pointed with **arrows**.

**FIGURE 7:**
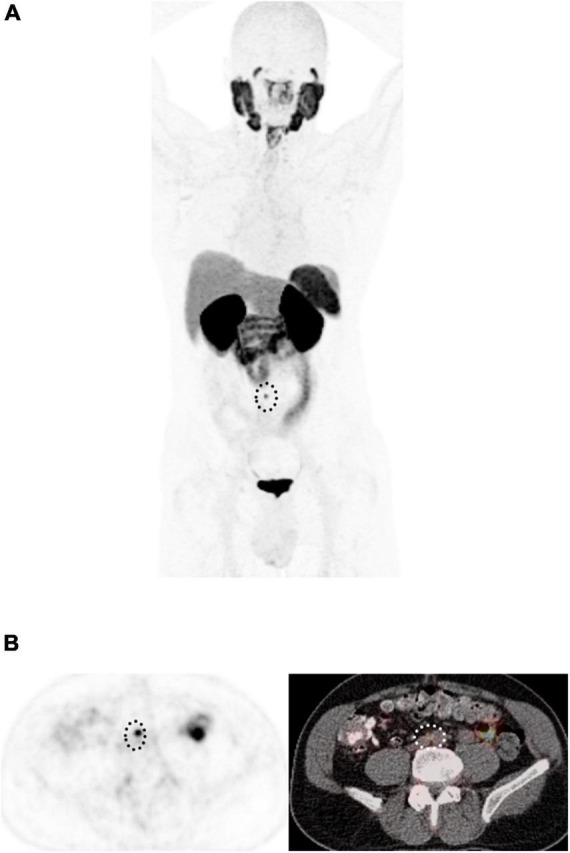
58-year-old man with biochemical recurrence of prostate cancer following radical prostatectomy. **(A)** Maximum intensity projection ^18^F-DCFPyL PET and **(B)** Axial PET (left) and fused PET/CT images show a single 5 mm PSMA-avid retroperitoneal lymph node (dotted circle), with miPSMA score of 2, PROMISE positive, in keeping with extraregional nodal recurrence.

The degree of PSMA uptake is qualitatively assessed in relation to uptake at reference tissues; mediastinal blood pool (MBP), liver and parotid gland. A molecular imaging PSMA score (miPSMA score) is assigned to each lesion, which can be null (score 0), low (score 1), intermediate (score 2) or high (score 3) as demonstrated in Figure 6. Lesions exhibiting high level of PSMA uptake (miPSMA score ≥ 2) are usually considered consistent with prostate cancer. As there are multiple different tracers available, is important to consider their specific biodistribution patterns when applying the classification; for example, for PSMA-targeted imaging with predominantly hepatobiliary excretion (e.g., ^18^F-PSMA-1007), the spleen is recommended as the intermediate reference instead of the liver (46). Once the score is assigned, it must be correlated with the appearance of the lesion in other modalities such as CT and MRI to provide the definitive diagnosis, as summarized in Table 3 and Figure 7.

### 2.3. Somatostatin-receptor PET in the staging of neuroendocrine tumors

#### 2.3.1. Krenning scoring system

**FIGURE 8:**
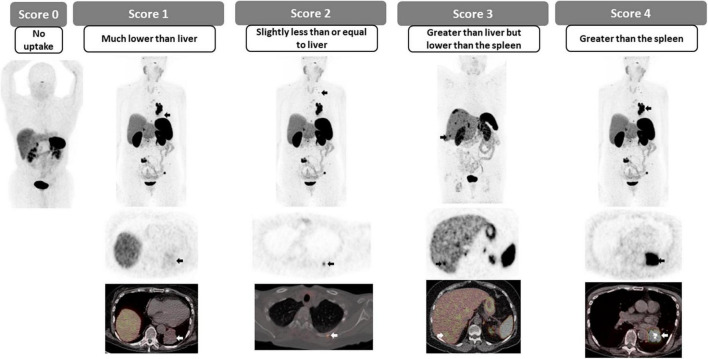
Krenning scoring system.

**FIGURE 9:**
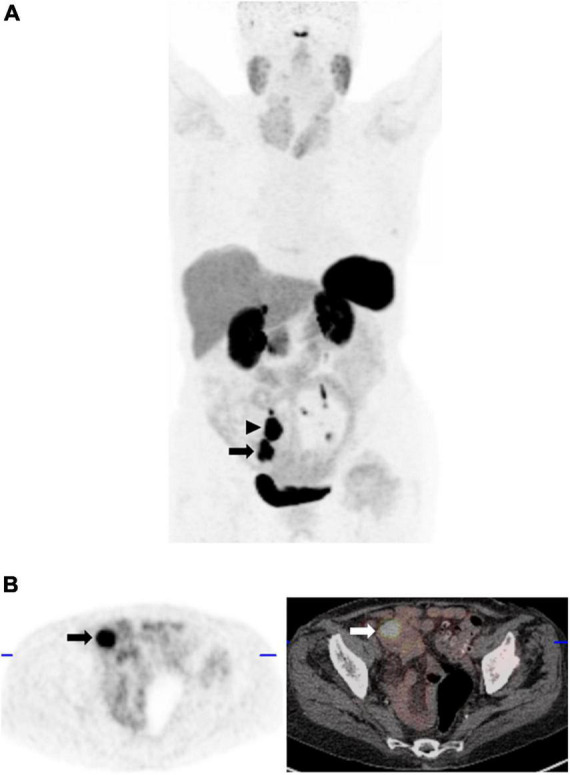
42-year-old man with clinical suspicion of small bowel NET. **(A)** Maximum intensity projection ^68^Ga-DOTATATE PET image shows 2 foci of intense abnormal ^68^Ga-DOTATATE uptake, greater than splenic uptake (Krenning score 4); the inferior corresponding to a mass in the terminal ileum (arrow), as well as a ^68^Ga-DOTATATE-avid mesenteric nodal mass (arrowhead) in keeping with an SSTR-2 overexpressing primary neuroendocrine tumor. **(B)** Corresponding axial PET (left) and fused PET/CT image at the level of the primary tumor (arrow).

The overexpression of somatostatin receptors (SSTR) on the cell surface of most neuroendocrine tumors has been exploited for imaging for a few decades, and more recently with ^68^Ga-(DOTA)-peptide PET (SSTR-PET). SSTR-PET has become a fundamental tool in the detection, staging and restaging of well-differentiated NET disease (47). The reported sensitivity of SSTR-PET for lesion detection has been reported to reach 96%, but this varies according to the size, location and SSTR expression. Furthermore, SSTR-PET has been shown to be superior to CT and SSTR-SPECT, providing additional information in up to 69% of cases (48–50). The Krenning scoring system, initially designed for scintigraphy (51), but recently adapted for PET imaging consists of a 5-point scale for the qualitative assessment of the degree of SSTRs overexpression. In this case, the uptake of the target lesion is compared to the uptake in reference tissues, the liver and the spleen or renal cortex in patients with prior splenectomy (Figures 8, 9). In patients with metastatic or non-resectable well-differentiated NETs, the overexpression of SSTR can be utilized for systemic radiotherapy delivery, in which case the peptide is labeled with an alpha or beta emitter to deliver radiotherapy to SSTR over-expressing targets; also known as peptide receptor radionuclide therapy (PRRT). The degree of SSTR over-expression is fundamental for appropriately selecting patients for PRRT, with a Krenning score of 3 or 4 indicating high degree of SSTR overexpression and suitability for PRRT in the appropriate clinical setting. In addition to directly correlating with response to PRRT, the phase III trial of 177Lu-DOTATATE for midgut NETs (NETTER-1) has shown that PRRT results in markedly longer PFS and higher response rates than high dose octreotide therapy (52). In patients with low Krenning scores, the presence of poorly differentiated or dedifferentiated tumor should be considered (and could be confirmed using dual imaging with ^18^F-FDG PET) and alternate systemic therapies should be considered (53, 54).

In conclusion, although not exhaustive, we have reviewed the common standardized reporting schemes used in clinical PET reporting in oncology. Applying scoring scales and reference tissues to assess radiotracer uptake qualitatively or semi quantitatively has been shown to increase reporting consistency with higher interobserver agreement in many clinical scenarios, often decreasing the frequency of indeterminate imaging findings. When validated, these interpretation schemes also provide a clinically usable reference to guide management; whether to de-escalate therapy in a patient with advanced Hodgkin’s lymphoma with a negative interim FDG PET after 2 cycles of chemotherapy, or to select patients with progressive, advanced midgut NET for PRRT. The more recently proposed interpretation schemes and/or implementation of existing schemes to new indications would require validation in further prospective studies before integration into routine clinical practice.

## Author contributions

VM and UM were responsible for material preparation, information collection and analysis, and prepared the manuscript. All authors contributed to the study conception and design and provided input of the content of the manuscript and approved the final version.

## References

[1] American College of Radiology. *Reporting and Data Systems (RADS).* (2022). Available online at: https://www.acr.org/Clinical-Resources/Reporting-and-Data-Systems (accessed November 30, 2022).

[2] Johnson P Federico M Kirkwood A Fosså A Berkahn L Carella A Adapted treatment guided by interim PET-CT scan in advanced Hodgkin’s lymphoma. *N Engl J Med.* (2016) 374:2419–29. 10.1056/NEJMoa1510093 27332902 PMC4961236

[3] Mehanna H Wong W McConkey C Rahman J Robinson M Hartley A PET-CT surveillance versus neck dissection in advanced head and neck cancer. *N Engl J Med.* (2016) 374:1444–54. 10.1056/NEJMoa1514493 27007578

[4] Meignan M Gallamini A Haiuon C. Report on the first international workshop on interim-PET scan in lymphoma. *Leuk Lymphoma.* (2009) 50:1257–60. 10.1080/10428190903040048 19544140

[5] Johnson S Kumar A Matasar M Schöder H Rademaker J. Imaging for staging and Response assessment in Lymphoma. *Radiology.* (2015) 276:323–38. 10.1148/radiol.2015142088 26203705

[6] Zamagni E Nanni C Dozza L Carlier T Bailly C Tacchetti P Standardization of 18 F-FDG-PET/CT according to deauville criteria for metabolic complete response definition in newly diagnosed multiple myeloma. *J Clin Oncol.* (2021) 39:116–25. 10.1200/JCO.20.00386 33151787

[7] Barrington S Kluge R. FDG PET for therapy monitoring in Hodgkin and non-Hodgkin lymphomas. *Eur J Nucl Med Mol Imaging.* (2017) 44 (Suppl. 1):97–110. 10.1007/s00259-017-3690-8 28411336 PMC5541086

[8] Cheson B Fisher R Barrington S Cavalli F Schwartz L Zucca E Recommendations for initial evaluation, staging, and response assessment of Hodgkin and non-Hodgkin lymphoma: the lugano classification. *J Clin Oncol.* (2014) 32:3059–68. 10.1200/JCO.2013.54.8800 25113753 PMC4979083

[9] Barrington SF Mikhaeel G Kostakoglu L Meignan M Hutchings M Müeller SP Role of imaging in the staging and response assessment of lymphoma: consensus of the international conference on malignant lymphomas imaging working group. *J Clin Oncol.* (2014) 32:3048–58. 10.1200/JCO.2013.53.5229 25113771 PMC5015423

[10] Gallamini A Zwarthoed C. Interim FDG-PET imaging in lymphoma. *Semin Nucl Med.* (2018) 48:17–27. 10.1053/j.semnuclmed.2017.09.002 29195613

[11] Gallamini A Borra A. Role of PET in lymphoma. *Curr Treat Options Oncol.* (2014) 15:248–61. 10.1007/s11864-014-0278-4 24619427

[12] Cronin C Swords R Truong M Viswanathan C Rohren E Giles F Clinical utility of PET/CT in lymphoma. *Am J Roentgenol.* (2010) 194:W91–103. 10.2214/AJR.09.2637 20028897

[13] Nols N Mounier N Bouazza S Lhommel R Costantini S Vander Borght T. Quantitative and qualitative analysis of metabolic response at interim positron emission tomography scan combined with international prognostic index is highly predictive of outcome in diffuse large B-cell lymphoma. *Leuk Lymphoma.* (2014) 55:773–80. 10.3109/10428194.2013.831848 23927393

[14] Straus D Jung S Pitcher B Kostakoglu L Grecula J Hsi E CALGB 50604: risk-adapted treatment of nonbulky early-stage Hodgking lymphoma based on interim PET. *Blood.* (2018) 132:1013–21. 10.1182/blood-2018-01-827246 30049811 PMC6128083

[15] Spaepen K Stroobants S Dupont P Van Steenweghen S Thomas J Vandenberghe P. Prognostic value of positron emission tomography (PET) with fluorine-18 fluorodeoxyglucose ([18F]FDG) after first-line chemotherapy in non-Hodgkin’s lymphoma: is [18F]FDG-PET a valid alternative to conventional diagnostic methods? *J Clin Oncol.* (2001) 19:414–9. 10.1200/JCO.2001.19.2.414 11208833

[16] Barnes JA LaCasce AS Zukotynski K Israel D Feng Y Neuberg D. End-of-treatment but not interim PET scan predicts outcome in nonbulky limited-stage Hodgkin’s lymphoma. *Ann Oncol.* (2011) 22:910–5. 10.1093/annonc/mdq549 20952598

[17] Moskowitz A Yahalom J Kewalramani T Maragulia J Vanak J Zelenetz A. Pretransplantation functional imaging predicts outcome following autologous stem cell transplantation for relapsed and refractory Hodgkin lymphoma. *Blood.* (2010) 116:4934–7. 10.1182/blood-2010-05-282756 20733154 PMC3799204

[18] Alcantara M Dupuis J Mareschal S Julian A Cottereau AS Becker S. PET/CT before autologous stem cell transplantation predicts outcome in refractory/relapsed follicular lymphoma. *Eur J Nucl Med Mol Imaging.* (2015) 42:215–21. 10.1007/s00259-014-2896-2 25239490

[19] Nanni C Versari A Chauvie S Bertone E Bianchi A Rensi M Interpretation criteria for FDG PET/CT in multiple myeloma (IMPeTUs): final results. IMPeTUs (Italian myeloma criteria for PET USe). *Eur J Nucl Med Mol Imaging.* (2018) 45:712–9. 10.1007/s00259-017-3909-8 29270787

[20] Zamagni E Patriarca F Nanni C Zanneti B Englaro E Pezzi A Prognostic relevance of 18-F FDG PET/CT in newly diagnosed multiple myeloma patients treated with up-front autologous transplantation. *Blood.* (2011) 118:5989–95. 10.1182/blood-2011-06-361386 21900189

[21] Usmani S Mitchel A Waheed S Crowley J Hoering A Barlogie B. Prognostic implications of serial 18-fluoro-deoxyglucose emission tomography in multiple myeloma treated with total therapy 3. *Blood.* (2013) 121:1819–23. 10.1182/blood-2012-08-451690 23305732 PMC3591801

[22] Zamagni E Nanni C Mancuso K Tacchetti P Pezzi A Pantani L PET/CT improves the definition of complete response and allows to detect otherwise unidentifiable skeletal progression in multiple myeloma. *Clin Cancer Res.* (2015) 21:4384–90. 10.1158/1078-0432.CCR-15-0396 26078390

[23] Sheikhbahaei S Taghipour M Ahmad R Fakhry C Kiess AP Chung C Diagnostic accuracy of follow-up FDG PET or PET/CT in patients with head and neck cancer after definitive treatment: a systematic review and meta-analysis. *Am J Roentgenol.* (2015) 205:629–39. 10.2214/AJR.14.14166 26295652

[24] Marcus C Ciarallo A Tahari A Mena E Koch W Wahl R Head and neck PET/CT: therapy response interpretation criteria (Hopkins Criteria) – interreader reliability, accuracy, and survival outcomes. *J Nucl Med.* (2014) 55:1411–6. 10.2967/jnumed.113.136796 24947059 PMC4390037

[25] Miller J Moradi F Sundaram V Liang R Zhang C Nguyen N Posttreatment FDG-PET/CT hopkins criteria predict locoregional recurrence after definitive radiotherapy for oropharyngeak squamous cell carcinoma. *Head Neck.* (2022) 44:2491–504. 10.1002/hed.27160 35920790 PMC12825993

[26] Kendi A Brandon D Switchenko J Wadsworth J El-Deiry M Saba N Head and neck PET/CT therapy response interpretation criteria (Hopkins criteria) – external validation study. *Am J Nucl Med Mol Imaging.* (2017) 7:174–80. 28913156 PMC5596320

[27] Zhong J Sundersingh M Dyker K Currie S Vaidyanathan S Prestwich R Post-treatment FDG PET-CT in head and neck carcinoma: comparative analysis of 4 qualitative interpretative criteria in a large patient cohort. *Sci Rep.* (2020) 10:4086. 10.1038/s41598-020-60739-3 32139722 PMC7058010

[28] Wang G Kurra V Gainor J Sullivan R Flaherty K Lee S Immune checkpoint inhibitor cancer therapy: spectrum of imaging findings. *Radiographics.* (2017) 37:2132–44. 10.1148/rg.2017170085 29131763

[29] Cheson B Ansell S Schwartz L Gordon LI Advani R Jacene HA Refinement of the lugano classification lymphoma response criteria in the era of immunomodulatory therapy. *Blood.* (2016) 128:2489–96. 10.1182/blood-2016-05-718528 27574190

[30] Ferrari C Maggialetti N Masi T Nappi AG Santo G Asabella A Early evaluation of immunotherapy response in lymphoma patients by 18F-FDG PET/CT: a literature overview. *J Pers Med.* (2021) 11:217. 10.3390/jpm11030217 33803667 PMC8002936

[31] Lee AJ Kim KW Cho YC Ko Y Sung YS Lee J Incidence of immune-mediated pseudoprogression of lymphoma treated with immune checkpoint inhibitors: systematic review and meta-analysis. *J Clin Med.* (2021) 10:2257. 10.3390/jcm10112257 34071024 PMC8197164

[32] Salles G Trotman J Lill M Cheson B Schuster S Hou J-Z Pseudo-progression among patients with follicular lymphoma treated with ibrutinib in the phase 2 DAWN study. *Blood.* (2016) 128:2980. 10.1182/blood.V128.22.2980.2980

[33] Tabaa YA Casasnovas O Baillet C Bachy E Virelizier N Schiano JM Prospective evaluation fo lymphoma response to immunomodulatory therapy criteria (LYRIC) in GATA trial from the LYSA group. *Hematol Oncol.* (2021) 39:5222. 10.1002/hon.157_2880

[34] Unterrainer M Ruzicka M Fabritius M Mittlmeier L Winkelmann M Rübenthaler J PET/CT imaging for tumour response assessment to immunotherapy: current status and future directions. *Eur Radiol Exp.* (2020) 4:2–13. 10.1186/s41747-020-00190-1 33200246 PMC7669926

[35] Wever E Maus M Mackall C. The emerging landscape of immune cell therapies. *Cell.* (2020) 181:46–62. 10.1016/j.cell.2020.03.001 32243795 PMC8900215

[36] Young H Baum R Cremerius U Herholz K Hoekstra O Lammertsma AA Measurement of clinical and subclinical tumour response using [^18^F]-fluorodeoxyglucose and positron emission tomography: review and 1999 EORTC recommendations. European organization for research and treatment of cancer (EORTC) PET study group. *Eur J Cancer*. (1999) 35:1773–82. 10.1016/s0959-8049(99)00229-4 10673991

[37] Wahl RL Jacene H Kasamon Y Lodge MA. From RECIST to PERCIST: evolving considerations for PET response criteria in solid tumors. *J Nucl Med*. (2009) 50:122S–50S. 10.2967/jnumed.108.057307 19403881 PMC2755245

[38] Cho S Lipson E Im H-J Rowe S Mena-Gonzalez E Blackford A Prediction of response to immune checkpoint inhibitor therapy using early time-point FDG-PET/CT imaging in patients with advanced melanoma. *J Nucl Med.* (2017) 58:1421–8. 10.2967/jnumed.116.188839 28360208 PMC5577627

[39] Anwar H Sachpekidis C Winkler J Kopp-Schneider A Haberko U Hassel J Absolute number of new lesions on 18 F-FDG PET/CT is more predictive of clinical response than SUV changes in metastatic melanoma patients receiving ipilimumab. *Eur J Nucl Med Mol Imaging.* (2018) 45:376–83. 10.1007/s00259-017-3870-6 29124281

[40] Sachpekidis C Anwar H Winkler J Kopp-Schneider A Larribere L Haberkorn U The role of interim 18F-FDG PET/CT in prediction of response to ipilimumab treatment in metastatic melanoma. *Eur J Nucl Med Mol Imaging.* (2018) 45:1289–96. 10.1007/s00259-018-3972-9 29478079

[41] Eiber M Herrmann K Calais J Hadaschik B Giesel F Hartenbach M Prostate cancer molecular imaging standardized evaluation (PROMISE): proposed miTNM classification for the Interpretation of PSMA-ligand PET/CT. *J Nucl Med.* (2018) 59:469–78. 10.2967/jnumed.117.198119 29123012

[42] Basso A Finelli A Bauman G Veit-Haibach P Berlin A Ortega C Impact of 18F-DCFPyL PET on staging and treatment of unfavorable intermediate or high-risk prostate cancer. *Radiology.* (2022) 24:211836. 10.1148/radiol.211836 35608445

[43] Fanti S Goffin K Hadaschik B Herrmann K Maurer T MacLennan S Consensus statements on PSMA PET/CT response assessment criteria in prostate cancer. *Eur J Nucl Med Mol Imaging.* (2021) 48:469–76. 10.1007/s00259-020-04934-4 32617640 PMC7835167

[44] Metser U Zukotynski K Mak V Langer D MacCrostie P Finelli A Effect of 18F-DCFPyL PET/CT on the management of patients with recurrent prostate cancer: results of a prospective multicenter registry trial. *Radiology.* (2022) 303:414–22. 10.1148/radiol.211824 35076300

[45] Cordula J Drendel V Rischke HC Beck T. Diagnostic accuracy of Ga-68-HBED-CC-PSMA-ligand-PET/CT before salvage lymph node dissection for recurrent prostate cancer. *Theranostics.* (2017) 7:1770–80. 10.7150/thno.18421 28529650 PMC5436526

[46] Giesel F Hadaschik B Cardinale J Radtke J Vinsensi M Lehnert W F-18 labelled PSMA-1007: biodistribution, radiation dosimetry and histopathological validation of tumor lesions in prostate cancer patients. *Eur J Nucl Med Mol Imaging.* (2017) 44:678–88. 10.1007/s00259-016-3573-4 27889802 PMC5323462

[47] Hope TA Bergsland EK Bozkurt MF Graham M Heaney A Herrmann K Appropriate use criteria for somatostatin receptor PET imaging in neuroendocrine tumors. *J Nucl Med.* (2018) 59:66–74. 10.2967/jnumed.117.202275 29025982 PMC6910630

[48] Koopmans K de Vries E Kema I Elsinga P Neels O Jager P. Staging of carcinoid tumours with 18FDOPA PET: a prospective, diagnostic accuracy study. *Lancet Oncol.* (2006) 7:728–34. 10.1016/S1470-2045(06)70801-4 16945767

[49] Hofman MS Kong G Neels OC Eu P Hong E Hicks RJ. High management impact of Ga-68 DOTATATE (GaTate) PET/CT for imaging neuroendocrine and other somatostatin expressing tumours. *Med Imaging Radiat Oncol.* (2012) 56:40–7. 10.1111/j.1754-9485.2011.02327.x 22339744

[50] Bozkurt M Virgolini I Balogova S Beheshti M Rubello D Decristoforo C Guideline for PET/CT imaging of neuroendocrine neoplasms with 68Ga-DOTA-conjugated somatostatin receptor targeting peptides and 18F–DOPA. *Eur J Nucl Med Mol Imaging.* (2017) 44:1588–601. 10.1007/s00259-017-3728-y 28547177

[51] Krenning E Bakker W Breeman W Koper J Kooij P Ausema L Localisation of endocrine-related tumours with radioiodinated analogue of somatostatin. *Lancet.* (1989) 333:242–4. 10.1016/S0140-6736(89)91258-0 2563413

[52] Strosberg J Haddad E Wolin E Hendifar A Yao B Chasen E Phase 3 trial of 177Lu-dotatate for midgut neuroendocrine tumors. *N Engl J Med.* (2017) 376:125–35. 10.1056/NEJMoa1607427 28076709 PMC5895095

[53] Chan D Pavlakis N Schembri G Bernard E Hsiao E Hayes A Dual somatostatin receptor/FDG PET/CT imaging in metastatic neuroendocrine tumours: proposal for a novel grading scheme with prognostic significance. *Theranostics.* (2017) 7:1149–58. 10.7150/thno.18068 28435454 PMC5399582

[54] Park S Parihar AS Bodei L Hope TA Mallak N Millo C Somatostatin receptor imaging and theranostics: current practice and future prospects. *J Nucl Med.* (2021) 62:1323–9. 10.2967/jnumed.120.251512 34301785 PMC9364764

