# Corrigendum: Standardized classification schemes in reporting oncologic PET/CT

**DOI:** 10.3389/fmed.2023.1324108

**Published:** 2023-11-01

**Authors:** Vanessa Murad, Roshini Kulanthaivelu, Claudia Ortega, Patrick Veit-Haibach, Ur Metser

**Affiliations:** ^1^Molecular Imaging Division, Joint Department of Medical Imaging, University Health Network, Mount Sinai Hospital and Women's College Hospital, University of Toronto, Toronto, ON, Canada; ^2^University Medical Imaging Toronto, University of Toronto, Toronto, ON, Canada

**Keywords:** PET, Deauville, Krenning, PROMISE, immunotherapy, head and neck cancer

In the published article, there was an error regarding the definition of disease progression according to PERCIMT criteria.

A correction has been made to **2. Reporting schemes and classifications**, *2.1. Therapy response assessment with 18F-FDG PET, 2.1.4. PET Response Evaluation Criteria for Immunotherapy (PERCIMT)*, paragraph 2.

This sentence previously stated:

“Disease progression is determined in the following 3 scenarios: (1) when there are 4 or more new lesions measuring less than 10 mm in their functional diameter, (2) when there are 3 or more new lesions also with functional diameter below 10 mm, or (3) when there are 2 or more new lesions with a functional diameter greater than 15 mm.”

The corrected sentence appears below:

“Disease progression is determined in the following 3 scenarios: (1) when there are 4 or more new lesions measuring less than 10 mm in their functional diameter, (2) when there are 3 or more new lesions with functional diameter greater than 10 mm, or (3) when there are 2 or more new lesions with a functional diameter greater than 15 mm.”

The authors apologize for this error and state that this does not change the scientific conclusions of the article in any way. The original article has been updated.

